# A Novel Presentation of Stanford Type A Aortic Dissection with Vaginal Bleeding: A Case Report

**DOI:** 10.5811/cpcem.47929

**Published:** 2025-10-21

**Authors:** Vijay Chandramaniya, Sanjay Mehta, Nandkishore Kapadia, Harikrishnan Chandramohanan, Jasmin Custodio

**Affiliations:** * Kokilaben Dhirubhai Ambani Hospital and Medical Research Institute, Department of Emergency Medicine, Mumbai, India; † Criticare Hospital, Department of Cardiovascular and Thoracic Surgery, Mumbai, India; ‡ Kiran Hospital, Heart-Lung Transplant Unit, Surat, Gujarat, India; § Krishna Vishwa Vidyapeeth, Department of Emergency Medicine, Karad, Maharashtra, India; ¶ Kern Medical, Department of Emergency Medicine, Bakersfield, California

**Keywords:** Stanford type A aortic dissection, aortic dissection, vaginal bleeding, case report

## Abstract

**Introduction:**

This case is unique in that it documents isolated, painless vaginal bleeding as the sole presenting symptom of a Stanford type A aortic dissection (STAAD), a presentation not previously reported. It adds to the literature by expanding the spectrum of atypical aortic dissection presentations and underscores the need to consider this diagnosis in elderly patients with vascular risk factors, even when they present with non-classical symptoms such as unexplained bleeding.

**Case Report:**

We present a novel case of STAAD in a 72-year-old woman with a history of hypertension, dyslipidaemia, prior hysterectomy, and cholecystectomy. Her primary complaint was a single, transient episode of painless vaginal bleeding. Notable clinical findings included a diminished right radial pulse, a significant inter-arm blood pressure discrepancy, and unremarkable systemic and vaginal examinations. Given these findings, further evaluation was pursued. Computed tomography aortography revealed a STAAD extending from the aortic arch to the bifurcation, involving the left internal iliac artery and a vaginal arterial branch. The patient underwent emergent surgical repair and was discharged in good condition on hospital day 11. At her most recent follow-up, three years post-event, she remained clinically stable with no recurrence.

**Conclusion:**

Isolated painless vaginal bleeding, although uncommon, may indicate life-threatening pathology. Subtle signs, such as inter-arm blood pressure discrepancy, can offer critical diagnostic clues, underscoring the importance of comprehensive evaluation in atypical emergency presentations.

## INTRODUCTION

Acute aortic dissection remains a diagnostic challenge in emergency medicine, particularly when presentations deviate from classical patterns. While sudden, severe chest, back, or abdominal pain is the hallmark symptom, up to 6% of patients—more often older women—present painlessly, leading to delays in recognition and intervention.^1^–^3^ Known atypical symptoms include neurologic deficits, syncope, and even gastrointestinal complaints, but isolated gynaecologic bleeding has not been previously documented. This diagnostic gap has implications for population-level outcomes, as early surgical management significantly reduces mortality.^4^

We present a novel case of Stanford type A aortic dissection (STAAD) in a 72-year-old woman whose only symptom was a single episode of painless vaginal bleeding. No previous reports in the literature describe this form of presentation. The case underscores how sex- and age-related atypical symptomatology can obscure time-critical diagnoses. It further highlights the importance of integrating subtle clinical cues, such as pulse deficits or blood pressure differentials, into a broad diagnostic framework, especially for high-risk patients. Recognizing such outliers is vital to ensuring equitable, life-saving care across diverse patient populations.

## CASE REPORT

A 72-year-old, moderately built female presented to the emergency department (ED) with a single, transient episode of painless vaginal bleeding that had occurred earlier that morning while bathing. She appeared mildly anxious, as she had not experienced any vaginal bleeding since undergoing a hysterectomy several years prior. She denied chest pain, back pain, abdominal or pelvic pain, as well as any history of coagulopathy, substance use, malignancy, or recent unintentional weight loss. She also reported no fever, urinary symptoms, vaginal rash or discharge, recent trauma, or haemorrhoids. Her medical history was significant for hypertension and dyslipidaemia. Surgical history included a hysterectomy for uterine fibroids and a cholecystectomy.

On physical examination, the patient was hemodynamically stable, with a respiratory rate of 16 breaths per minute and an oxygen saturation of 96% on room air. Her heart rate was 102 beats per minute. Cardiac examination revealed normal heart sounds without murmurs. Respiratory, neurological, abdominal, and pelvic examinations were unremarkable. All extremities were warm and well-perfused, with no evidence of cyanosis. Peripheral pulses were well-felt and symmetrical, except for a comparatively diminished right radial pulse, which was identified during routine pulse assessment performed on all patients in our ED, regardless of presenting complaint. Blood pressure in the right arm was 86/60 millimetres of mercury (mm Hg), a finding not explained by the patient’s single, transient episode of minimal vaginal bleeding. Given the unexplained hypotension and the subtle discrepancy in pulse volume between the arms, blood pressure was also measured in the left arm. A significant inter-arm blood pressure difference (reference range: <10 mm Hg) was noted: 86/60 mm Hg in the right arm and 160/80 mm Hg in the left—an abnormality that had not been previously reported or known to the patient. A vaginal examination performed by the gynaecologist was unremarkable and did not reveal any identifiable source of bleeding.

The diagnostic assessment began with a physical examination, which revealed a diminished right radial pulse and a significant inter-arm blood pressure discrepancy. The hypotension in the right arm was disproportionate to the transient, minimal vaginal bleeding and raised concern for an underlying alternative pathology. Despite the absence of chest, back, or abdominal pain, the findings raised concern for a vascular aetiology, particularly involving the aorta or peripheral vessels. Laboratory tests were unremarkable and did not suggest infection or coagulopathy. The patient’s medical history, along with a non-contributory vaginal examination performed by the gynaecologist, made local gynaecologic causes of vaginal bleeding less likely. Given the atypical presentation—painless vaginal bleeding in an elderly woman with cardiovascular risk factors—combined with abnormal vital signs, advanced imaging was pursued in consultation with vascular surgery. Computed tomography (CT) aortography confirmed a STAAD extending from the aortic arch to the aortic bifurcation, with further extension into the left internal iliac artery and one of the vaginal arterial branches (Image).

*CPC-EM Capsule*

What do we already know about this clinical entity?

*Aortic dissection may present without pain; atypical cases in elderly women often face delayed diagnosis and high mortality*.

What makes this presentation of disease reportable?

*Isolated painless vaginal bleeding as the sole manifestation of Stanford type A aortic dissection is novel and unreported in the medical literature*.

What is the major learning point?

*Subtle findings such as inter-arm blood pressure difference can uncover life-threatening vascular disease, even without classic symptoms*.

How might this improve emergency medicine practice?

*Maintaining a high suspicion for aortic dissection in atypical cases can expedite diagnosis and enable timely, life-saving treatment*.

The primary diagnostic challenge was the absence of classic symptoms of aortic dissection, a factor that can contribute to delayed diagnosis, particularly in older women. The source of bleeding was also highly atypical, initially suggesting differential diagnoses such as gynaecologic bleeding of local origin (eg, vaginal cuff granulation or neoplasia), coagulopathy, or vascular malformations. However, these possibilities were inconsistent with the patient’s medical history, stable laboratory results, and normal physical examination findings. In contrast, careful attention to the vascular exam guided the diagnostic approach toward the correct diagnosis. Following early recognition, the prognosis improved significantly with prompt surgical intervention. Aortic dissection, particularly type A dissections, carries a high mortality rate if left untreated, but early diagnosis and repair substantially improve outcomes.

The patient underwent emergent surgical repair, including reconstruction of the sinus of Valsalva, ascending aorta replacement, and hemi-arch repair. Her postoperative course was notable for a left-sided pneumothorax on day 3, which was promptly managed with intercostal drain placement and resolved without further complication. She was discharged in stable condition on postoperative day 11. Twenty days after discharge, she was hospitalized for new-onset atrial fibrillation, which was managed conservatively with oral anti-arrhythmic medications and sinus rhythm maintained without recurrence. At her most recent follow-up, three years after the event, the patient remained clinically stable without new symptoms, complications, or recurrence.

## DISCUSSION

The timely recognition of an atypical presentation of STAAD enabled life-saving intervention. Despite the absence of classic symptoms, the presence of a significant inter-arm blood pressure discrepancy—combined with the lack of an identifiable alternative source for the vaginal bleeding—prompted a high index of suspicion and led to appropriate diagnostic imaging. (We were unable to definitively confirm the vascular source of the vaginal bleeding due to the urgency of surgical intervention and the absence of tissue-level vascular mapping.)

Aortic dissection is a time-sensitive vascular emergency with high early mortality. Without treatment, AAD carries an estimated mortality rate of approximately 50% within the first 48 hours.^1^ However, timely surgical intervention significantly reduces this risk. Data from the International Registry of Acute Aortic Dissection (IRAD) demonstrated a 48-hour mortality of only 4.4% with prompt surgery.^4^ Historically, atypical presentations of STAAD have contributed to delayed diagnoses. According to Harris et al, AAD is less frequently observed in older females and is more likely to present with nonspecific or neurological symptoms.^5^

Our case contributes to the expanding literature on unusual manifestations of STAAD. While previous reports have described atypical presentations of AAD—including painless paralysis,^8^ sore throat,^9^ testicular pain,^11^ symptoms mimicking ureteral calculus,^10^ pulmonary embolism^12^ or bowel obstruction,^14^ and an elderly, hypertensive female presenting with profound weakness and hypotension^15^—vaginal bleeding has not been previously documented as a presenting feature. In this case, CT aortography revealed dissection extending into the left internal iliac artery and a vaginal arterial branch, offering a plausible anatomical basis for the bleeding (Figure).

Although a significant systolic blood pressure differential between arms is a known sign of AAD, its diagnostic accuracy is limited.^6^ Nonetheless, in our patient, this finding prompted further imaging that confirmed the diagnosis. As IRAD data and subsequent studies have shown, early recognition and intervention can drastically alter the prognosis of STAAD.^3^,^4^,^7^

## CONCLUSION

In this case presentation, the aortic dissection was the likely cause of the patient’s vaginal bleeding, rather than a coincidental finding, based on several key factors. First, a thorough pelvic examination performed by a gynaecologist was entirely normal, with a recommendation to investigate causes beyond the local genital tract. Second, the patient had no prior medical history suggestive of a cause for vaginal bleeding, and her laboratory parameters were within normal limits. Third, a newly identified significant inter-arm blood pressure discrepancy, in the context of long-standing hypertension, raised suspicion for a vascular aetiology. The aortic dissection may have initiated atypically and progressed in a manner that involved extension of the intimal flap into a branch supplying the vaginal vasculature. This could have led to a minor arterial or pseudo-aneurysmal rupture, followed by localized thrombosis, resulting in a single episode of minimal vaginal bleeding.

## PATIENT PERSPECTIVE

The patient expressed gratitude for the timely and thorough care she received. She was surprised that a serious cardiac condition could present as vaginal bleeding but felt reassured by the emergency team’s evaluation. Despite a challenging recovery, she remains committed to ongoing treatment and follow up.

## Figures and Tables

**Figure:**
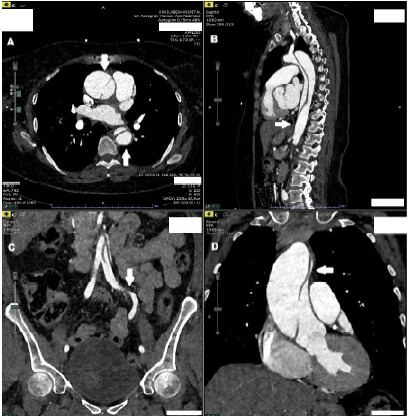
Focused illustration of vaginal vascular anatomy. The diagram illustrates the descending aorta branching into the common iliac, internal iliac, and subsequently the vaginal artery, which supplies the vaginal canal. This schematic illustrates the anatomical context of potential vascular involvement in cases of aortic dissection presenting with vaginal bleeding.

**Image:**
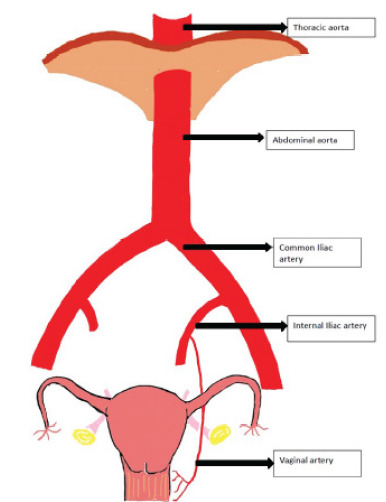
Axial (A), sagittal (B), and coronal (C, D) computed tomography aortography views demonstrating Stanford type A aortic dissection. Panel A shows a dissection flap (arrow) in the ascending aorta and aortic arch. Panel B reveals the dissection flap (arrow) extending through the thoracic aorta in the sagittal view. Panel C shows involvement of the abdominal aorta (arrow), with propagation of the dissection flap inferiorly into the left common iliac artery. Panel D shows the origin of the dissection flap (arrow) in the proximal ascending aorta in coronal reconstruction.
